# Flower visitation by hoverflies (Diptera: Syrphidae) in a temperate plant-pollinator network

**DOI:** 10.7717/peerj.6025

**Published:** 2018-12-03

**Authors:** Jan Klecka, Jiří Hadrava, Paolo Biella, Asma Akter

**Affiliations:** 1 Czech Academy of Sciences, Biology Centre, Institute of Entomology, České Budějovice, Czech Republic; 2 Department of Zoology, Faculty of Science, Charles University, Prague, Czech Republic; 3 Department of Zoology, Faculty of Science, University of South Bohemia, České Budějovice, Czech Republic

**Keywords:** Pollination, Plant-pollinator interactions, Foraging, Hoverflies, Species traits, Pollination networks

## Abstract

Hoverflies (Diptera: Syrphidae) are among the most important pollinators, although they attract less attention than bees. They are usually thought to be rather opportunistic flower visitors, although previous studied demonstrated that they show colour preferences and their nectar feeding is affected by morphological constraints related to flower morphology. Despite the growing appreciation of hoverflies and other non-bee insects as pollinators, there is a lack of community-wide studies of flower visitation by syrphids. The aim of this paper is to provide a detailed analysis of flower visitation patterns in a species rich community of syrphids in a Central European grassland and to evaluate how species traits shape the structure of the plant-hoverfly flower visitation network. We found that different species varied in the level of specialisation, and while some species visited a similar spectre of flowers, others partitioned resources more strongly. There was a consistent difference in both specialisation and flower preferences between three syrphid subfamilies. Eristalinae and Pipizinae were more specialised than Syrphinae. Trait-based analyses showed that relative flower visitation (i) increased with plant height, but most strongly in Eristalinae; (ii) increased with inflorescence size in small species from all three subfamilies, but was independent of inflorescence size in large species of Eristalinae and Syrphinae; and (iii) depended on flower colour, but in a subfamily-specific way. Eristalinae showed the strongest flower colour preferences for white flowers, Pipizinae visited mostly white and yellow flowers, while Syrphinae were less affected by flower colour. Exploration of the structure of the plant-hoverfly flower visitation network showed that the network was both modular and nested. We also found that there were almost no differences in specialisation and relative visitation frequency between males and females. Overall, we showed that flower visitation in syrphids was affected by phylogenetic relatedness, body size of syrphids and several plant traits.

## Introduction

Hoverflies (Diptera: Syrphidae) are one of the most abundant groups of flower visiting insects. Together with other families of flies, their role in plant–pollinator interactions is often underappreciated (Inouye et al., 2015). However, Diptera often make up a similar proportion of flower visitors as Hymenoptera and are even the dominant group of pollinators in some habitats, e.g., in higher altitudes and latitudes (Kanstrup & Olesen, 2000).

Hoverflies are important pollinators of many wild plants (Orford, Vaughan & Memmott, 2015; Sakurai & Takahashi, 2017; Moquet et al., 2018), in some cases as important as bees (Forup et al., 2008), and bringing the most pollen grains per visit to flowers of some plant species (King, Ballantyne & Willmer, 2013). They also play an important role in pollination of numerous crops (Solomon & Kendall, 1970; Kendall et al., 1971; Ohsawa & Namai, 1987; Ohsawa & Namai, 1988; Jauker & Wolters, 2008; Ssymank et al., 2008; Rader et al., 2009; Inouye et al., 2015; Rader et al., 2016). Hoverflies are thus an important group of pollinators not only from the perspective of biodiversity conservation, but also for pollination of crops. The interest in the role of flies in general and Syrphidae in particular as pollinators has been increasing (Ssymank et al., 2008; Lucas et al., 2018a; Lucas et al., 2018b). However, our knowledge of their preferences for different flowers and their partitioning of floral resources is still limited.

Adults of all known syrphid species feed almost exclusively on pollen and nectar or honeydew (Rotheray & Gilbert, 2011) and are usually considered to be generalist flower visitors (Branquart & Hemptinne, 2000; Lucas et al., 2018a). However, individual species cover a broad range from generalists to species with strong preferences for a small number of plants (Branquart & Hemptinne, 2000; Colley & Luna, 2000). Their flower preferences may, however, shift depending on local flower availability and plant phenology (Cowgill, Sotherton & Wratten, 1993; Colley & Luna, 2000), which can lead to changing levels of generalisation during the season (Lucas et al., 2018b). Several studies reported that selectivity of some hoverfly species depends on certain plant traits. Overall, hoverflies seem to visit mostly open bowl-shaped flowers (Branquart & Hemptinne, 2000), where they feed on both nectar and pollen (Gilbert, 1981), but some of them have relatively long proboscises which allow them to reach nectar even in flowers with relatively long spurs (Gilbert, 1981; Vlašánková et al., 2017). In addition, inflorescence height also affects flower visitation by some species (Gervasi & Schiestl, 2017; Klecka, Hadrava & Koloušková, 2017). It has been recently demonstrated that selective flower visitation by hoverflies can exert a selection pressure strong enough to cause rapid evolutionary shifts in multiple plant traits (Gervasi & Schiestl, 2017; Zu & Schiestl, 2017). However, there are still very few studies focusing on flower visitation patterns of entire local assemblages of syrphids.

Entire plant-flower visitor networks are characterised by distinct structural features related to biological constraints on interspecific interactions. Most plant-flower visitor networks are strongly nested (Bascompte et al., 2003), modular (Olesen et al., 2007), or both nested and modular at the same time (Fortuna et al., 2010). Modules pack species connected by numerous interactions, at least in some cases based on trait complementarity between the interacting partners (Olesen et al., 2007; Carstensen, Sabatino & Morellato, 2016; Dormann, Fründ & Schaefer, 2017). On the other hand, nested structure means that specialised insects tend to interact with plants also visited by more generalised insects and vice versa. In this case, mutual specialisation is rare, which has a stabilising effect on the structure of plant-flower visitor networks (Burgos et al., 2007; Bastolla et al., 2009).

The aim of this paper is to advance our understanding of flower visitation by hoverflies by a thorough analysis of flower visitation in a species-rich community of plants in a Central European grassland. Specifically, we tested whether different species exhibit various levels of specialisation, whether there are consistent differences between species from three hoverfly subfamilies, and whether males and females of individual species have distinct preferences for flowers. We also tested whether plant species traits, such as inflorescence height, size, and flower colour affect flower visitation by hoverflies from different subfamilies and of different body sizes. Finally, we explored the structural features of the entire plant-hoverfly network.

## Methods

**Figure 1:**
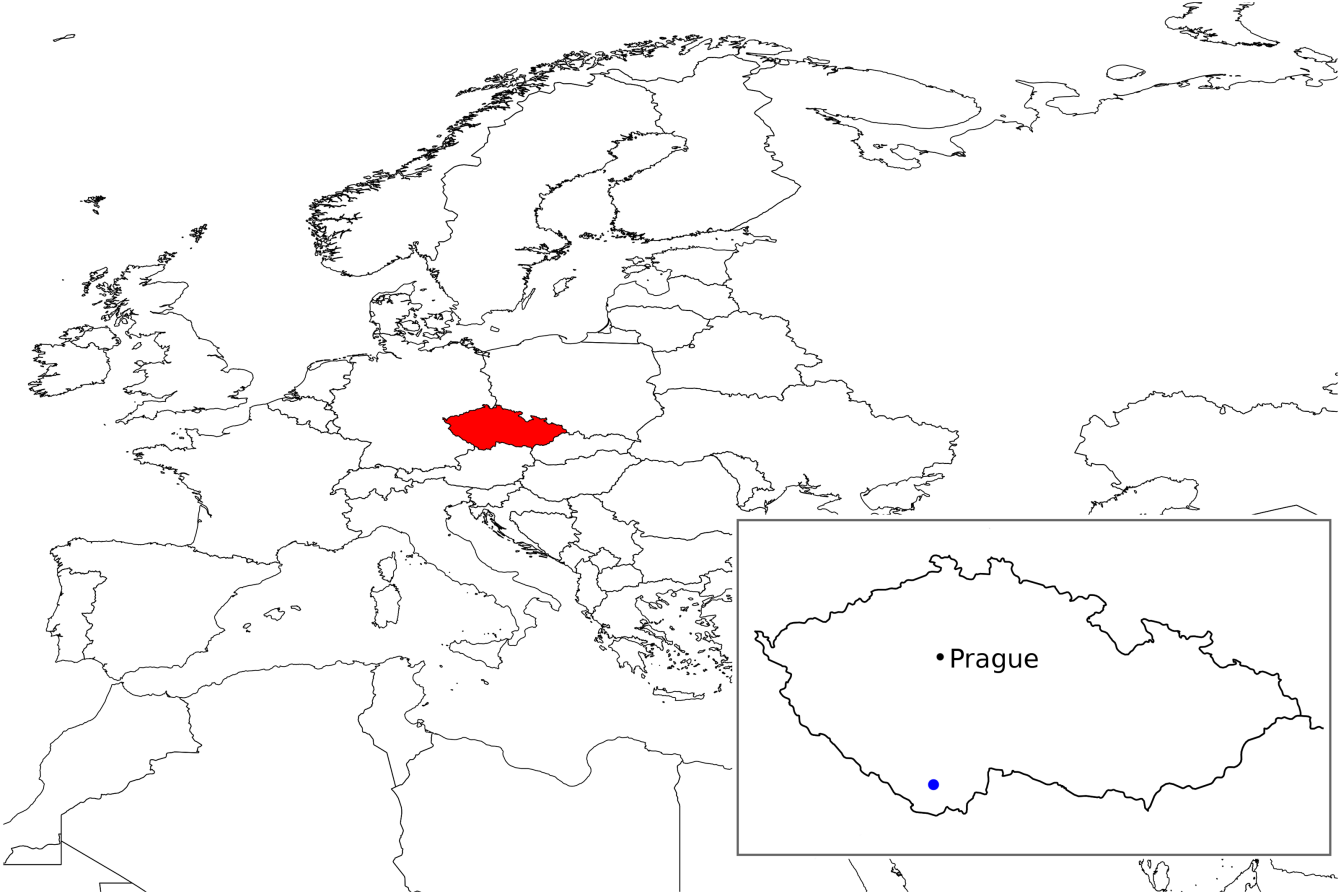
The location of the study area. The study was conducted in the southern part of the Czech Republic, the location of the study area is shown in the inset by a blue circle. The map was plotted using the rworldmap package in R (South, 2011).

We conducted sampling of plant-flower visitor interactions in a small area (ca 2 km^2^) in the southern part of the Czech Republic (Fig. 1) between the northern edge of the city of Český Krumlov and nearby villages Vyšný and Lazec in June-August 2015. We gathered observations from eight small (<1 ha), flower-rich grassland patches partly surrounded by shrubs and trees between 48°49′29.5″N, 14°18′59.5″E in the South, 48°49′42.6″N, 14°19′24.4″E in the East, and 48°50′7.0″N, 14°15′36.5″E in the North–West. Geographic coordinates of each sample are included in Table S1. The study area was relatively dry, partly calcareous. The patches where we sampled were only extensively managed, partly by occasional grazing by cows or sheep, partly by infrequent mowing. Among the most abundant flowering plants were *Centaurea scabiosa*, *Galium mollugo*, *Galium verum*, *Agrimonia eupatoria*, and *Daucus carota*. Sampling was conducted on public land and did not involve any protected species. For this reason, we did not need to obtain any permits for this project.

We sampled flower-visiting insects by transect walks and collected all visitors to flowers of all herbaceous flowering plants except grasses, although some syrphids are known to feed on pollen of grasses and sedges (Ssymank & Gilbert, 1993). We sampled six short (10 m long and 1 m wide) transects in each pacth during multiple visits between 25 June and 1 August 2015. We walked along the transect slowly and captured all flower visiting insects from all flowers. Sampling was carried out during sunny days with no rain between 08:00 and 17:00 h. We designed this sampling strategy to gather standardised quantitative data on flower visitation representative of the local plant community. In this paper, we focus on hoverflies (Diptera: Syrphidae), which were among the most abundant groups of flower visiting insects in the study area. All collected individuals were killed by ethyl-acetate, transported to the lab, pinned, and identified using keys of Van Veen (2010) and Speight & Sarthou (2014) to the species level. Identification of hoverflies of the genus *Pipizella* and a few damaged specimens of *Platycheirus* was verified by DNA barcoding using the 5′ region of the COI gene. We also identified the sex of all individuals. Several individuals per species were photographed using a stereomicroscope Olympus SZX7 and a DSLR camera Canon 700D controlled from a computer by digiCamControl software. We measured body length, thorax width, and head width of at least 8 individuals, or all individuals in species where less individuals were collected, using the Fiji distribution (Schindelin et al., 2012) of ImageJ (Rueden et al., 2017) and a plugin Microscope Measurement Tools. All specimens are deposited in Jan Klecka’s collection at the Institute of Entomology, Biology Centre of the Czech Academy of Sciences.

We measured a set of three traits of all plant species visited by hoverflies to test their effect on flower visitation (Table S4). Specifically, we measured plant height (the height of the top flower above ground), inflorescence size (the largest distance between any two open flowers within an inflorescence), and classified flower colour into four categories (blue, purple, white, and yellow), similarly to previous studies (Haslett, 1989a). We conducted plant trait measurements in several locations across the study area for each species, measuring at least 10 plant individuals in each sampled patch, with the exception of very rare species.

For data analysis, we pooled observations from the entire study area, because most sampled patches were close to each other (<1 km apart), well within dispersal range of most hoverflies (Rader et al., 2011; Moquet et al., 2018). We performed all analyses in R 3.2.3 (R Core Team, 2015). We visualised the structure of plant-hoverfly flower visitation network using the package bipartite 2.08 (Dormann et al., 2009; Dormann, 2011). To measure specialisation of individual species, we calculated the specialisation index *d*′ according to Blüthgen, Menzel & Blüthgen (2006). We used generalised linear models (GLM) for most statistical analyses, either with a Poisson distribution with overdispersion (quasiPoisson), a Binomial distribution with overdispersion (quasibinomial), or Normal distribution depending on the properties of the response variable. We used non-metric multidimensional scaling implemented in vegan 2.4-4 package for R (Oksanen et al., 2017) to visualise diet overlap between syrphid species. Diet overlap was calculated using Pianka’s overlap index (Pianka, 1973) using the plant-hoverfly flower visitation matrix (Table S2) to estimate pairwise diet overlap values between all pairs of hoverfly species. We performed the diet overlap analysis using EcoSimR 0.1.0 package for R (Gotelli, Hart & Ellison, 2015). Finally, we performed analyses of modularity and nestedness of the plant-hoverfly flower visitation network using the package bipartite 2.08 for R (Dormann et al., 2009; Dormann, 2011).

## Results

**Figure 2:**
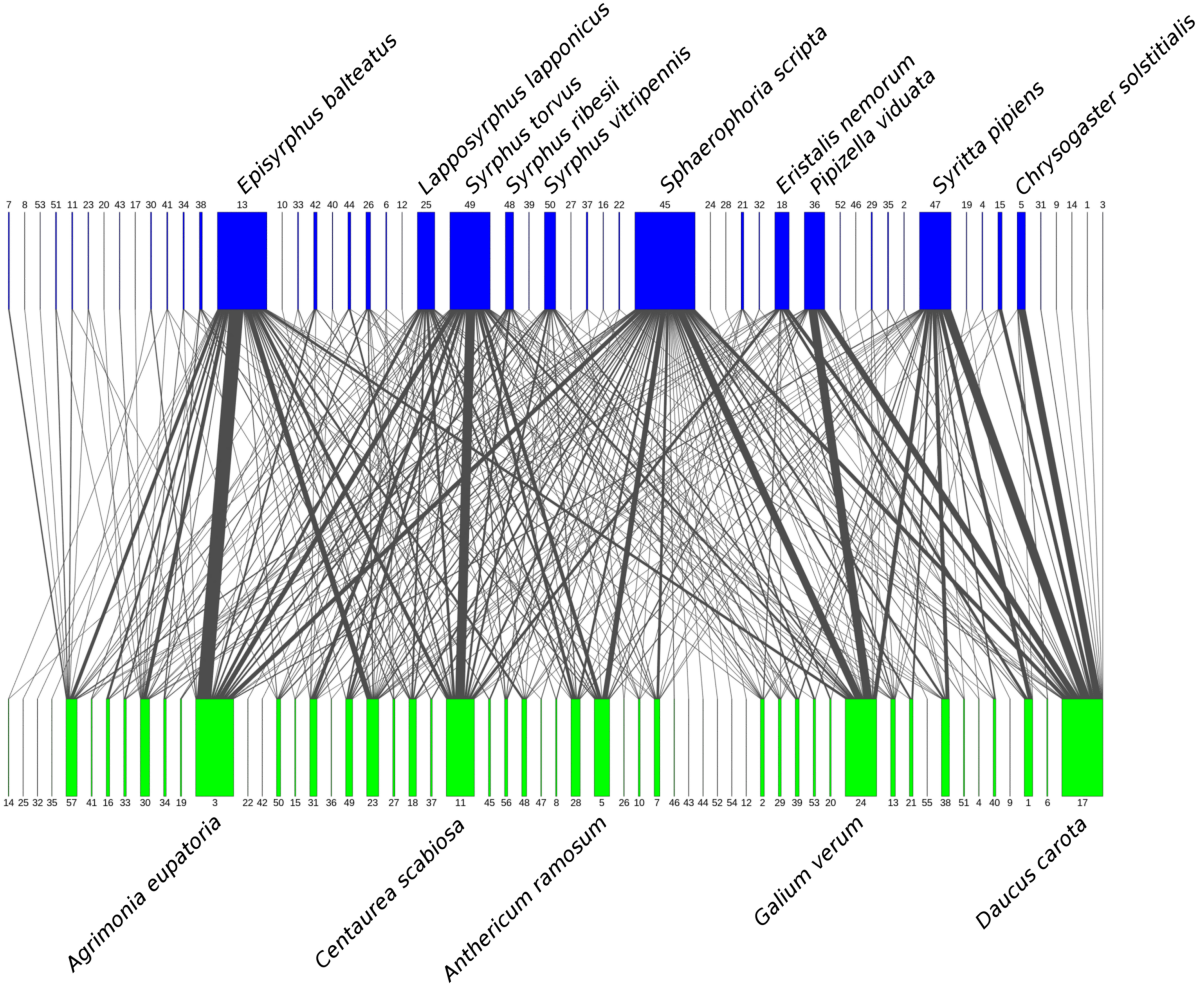
Flower visitation network of plants and hoverflies. Hoverflies are displayed in the upper row with blue boxes. The width of the boxes is proportional to the number of individuals observed. Plants are displayed in the lower row as green boxes whose width is proportional to the number of flower visits observed. The width of the connecting lines is proportional to the number of interactions observed between each plant-syrphid pair. The most abundant species are named, all species are identified by numbers—see Table S2 for legend.

We observed 1194 interactions between a total of 53 species of syrphids from three subfamilies (Eristalinae, Pipizinae, and Syrphinae) and 57 plant species from 20 families (Table 1). The network of plant-syrphid flower visiting interactions is shown in Fig. 2, raw data are available in Tables S1, S2.

**Figure 3:**
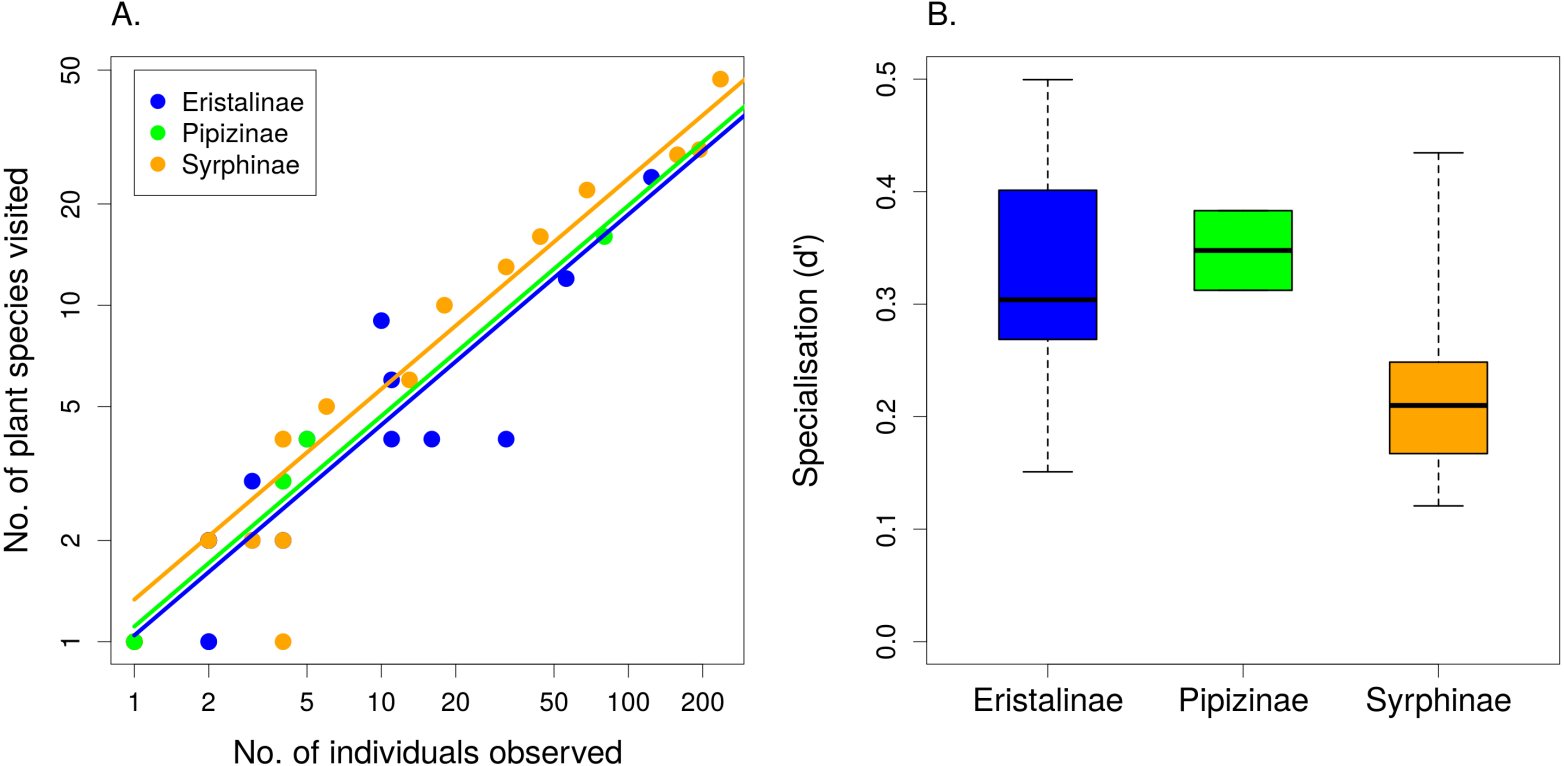
The level of resource specialisation of hoverflies. (A) The number of plant species visited depended on the number of observations. Variation around the regression line shows that species below the line were more specialised than expected and species above the line were more generalised. (B) Syrphinae were more generalised than Eristalinae and Pipizinae (mean, quartiles, and range shown).

The number of plant species visited by individual syrphid species increased with the number of observations linearly on a log–log scale (*F*_1,49_ = 986.49, *P* < 1∗10^−6^) and differed significantly between subfamilies, with Syrphinae visiting more plant species than Eristalinae and Pipizinae after accounting for the number of observations (*F*_2,49_ = 4.32, *P* = 0.0187; Fig. 3A). Species from the subfamily Syrphinae were more generalised than the other two subfamilies also according to our calculation of a specialisation index *d*′ (*F*_2,16_ = 3.733, *P* = 0.0467; Fig. 3B), which was restricted to species with at least five observations. Body length had no effect on the number of plant species visited (*F*_1,47_ = 0.47, *P* = 0.4986), nor on the value of the specialisation index *d*′ (*F*_1,15_ = 1.44, *P* = 0.2484). The same results were obtained using head width and thorax width as alternative measures of body size. Comparison of the specialisation index *d*′ revealed no consistent differences in specialisation between males and females (linear mixed effects model with species as a random factor; ${\chi }_{1}^{2}=0.93$, *P* = 0.3362) (Table 2).

**Table 1:** The list of species of Syrphidae collected during this study. The number of observations and the number of plant species visited is provided for each species and sex together with mean body length for each species.

	No. of observations			No. of plant species visited			Body length (mm)	Head width (mm)
	Total	Females	Males	Total	Females	Males		
**Eristalinae**								
*Cheilosia proxima*	1	0	1	1	0	1	9.55	2.90
*Cheilosia ruficollis*	2	2	0	2	2	0	6.84	2.30
*Chrysogaster basalis*	1	1	0	1	1	0	8.56	2.68
*Chrysogaster coemiteriorum*	3	3	0	2	2	0	7.95	2.86
*Chrysogaster solstitialis*	32	20	12	4	2	3	7.39	2.39
*Eristalinus sepulchralis*	1	0	1	1	0	1	7.46	2.97
*Eristalis arbustorum*	16	5	11	4	2	3	10.53	3.73
*Eristalis horticola*	2	2	0	2	2	0	11.90	4.53
*Eristalis intricaria*	1	0	1	1	0	1	12.33	4.42
*Eristalis nemorum*	56	32	24	12	10	8	11.90	4.12
*Eristalis pertinax*	2	1	1	2	1	1	13.98	4.52
*Eristalis similis*	1	0	1	1	0	1	14.21	4.85
*Eristalis tenax*	10	8	2	9	8	2	14.52	5.30
*Helophilus pendulus*	3	1	2	3	1	2	12.12	3.96
*Myathropa florea*	5	3	2	4	2	2	12.09	4.20
*Neoascia podagrica*	4	4	0	2	2	0	4.83	1.31
*Orthonevra nobilis*	2	1	1	1	1	1	5.35	1.71
*Rhingia campestris*	11	4	7	6	3	6	8.79	3.04
*Rhingia rostrata*	2	0	2	2	0	2	7.31	2.44
*Sericomyia bombiformis*	2	0	2	1	0	1	15.55	4.99
*Sericomyia silentis*	11	6	5	4	3	3	14.75	5.10
*Sphegina sp*	1	1	0	1	1	0	6.54	1.34
*Syritta pipiens*	124	72	52	24	18	16	7.86	1.97
*Volucella bombylans*	4	4	0	2	2	0	13.07	4.84
*Volucella pellucens*	2	2	0	2	2	0	14.68	5.25
**Pipizinae**								
*Heringia pubescens*	1	1	0	1	1	0	5.74	2.00
*Pipiza noctiluca*	5	5	0	4	4	0	8.94	2.51
*Pipizella annulata*	4	2	2	3	2	1	6.67	2.14
*Pipizella viduata*	80	39	41	16	9	10	5.62	1.79
**Syrphinae**								
*Chrysotoxum bicinctum*	4	3	1	4	3	1	10.54	3.32
*Chrysotoxum cautum*	4	4	0	1	1	0	13.41	4.48
*Chrysotoxum fasciatum*	1	1	0	1	1	0	12.26	4.15
*Chrysotoxum vernale*	1	1	0	1	1	0	12.77	3.93
*Chrysotoxum verralli*	1	0	1	1	0	1	11.72	3.79
*Didea alneti*	4	1	3	2	1	2	12.45	3.65
*Epistrophe grossulariae*	1	0	1	1	0	1	13.01	3.87
*Episyrphus balteatus*	194	91	103	29	23	22	9.79	2.63
*Eupeodes bucculatus*	4	1	3	4	1	3	8.85	2.76
*Lapposyrphus lapponicus*	68	23	45	22	12	17	10.20	3.03
*Melanostoma mellinum*	18	8	10	10	6	7	6.74	1.82
*Melanostoma scalare*	1	0	1	1	0	1	8.26	2.02
*Meliscaeva cinctella*	1	0	1	1	0	1	9.17	2.54
*Paragus haemorrhous*	3	2	1	2	2	1	5.46	1.71
*Parasyrphus lineolus*	4	2	2	3	2	2	9.23	2.80
*Platycheirus albimanus*	6	3	3	5	3	3	7.19	2.11
*Scaeva dignota*	2	1	1	2	1	1	12.06	3.61
*Scaeva pyrastri*	4	1	3	4	1	3	13.09	5.37
*Scaeva selenitica*	13	4	9	6	4	3	13.75	4.11
*Sphaerophoria scripta*	236	142	94	47	38	34	8.89	2.04
*Syrphus ribesii*	32	20	12	13	9	6	11.14	3.43
*Syrphus torvus*	158	70	88	28	21	21	10.77	3.42
*Syrphus vitripennis*	44	28	16	16	14	10	10.23	3.16
*Xanthogramma pedissequum*	1	1	0	1	1	0	12.07	3.39

**Figure 4:**
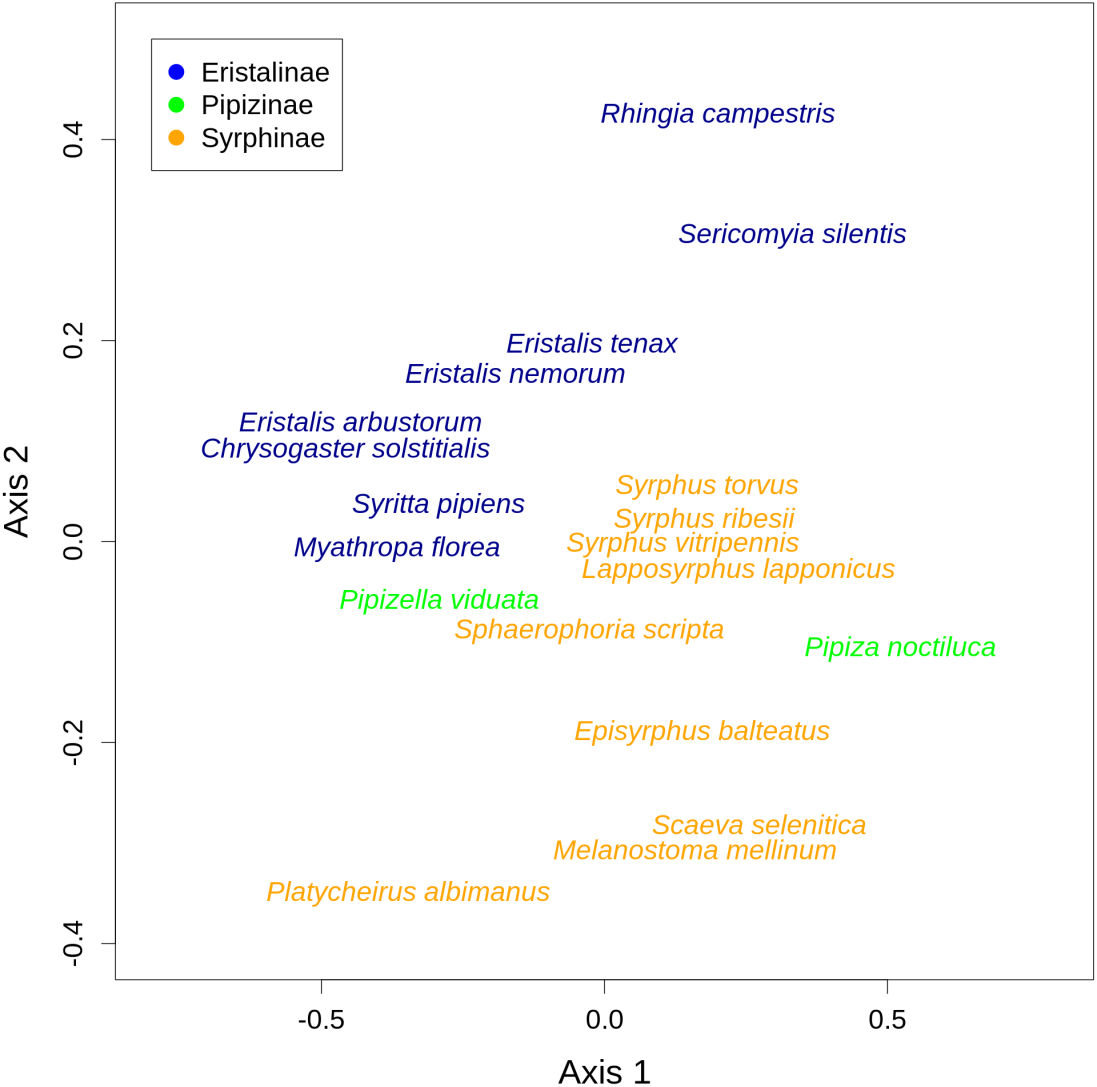
Results of nonmetric multidimensional scaling (NMDS) show differences in flower preferences in Syrphinae and Eristalinae. NMDS analysis was run with a matrix of dissimilarities of the relative frequency of flower visitation on different plants by individual species of syrphids. The position of individual species in the plot corresponds to the center of the species label.

**Figure 5:**
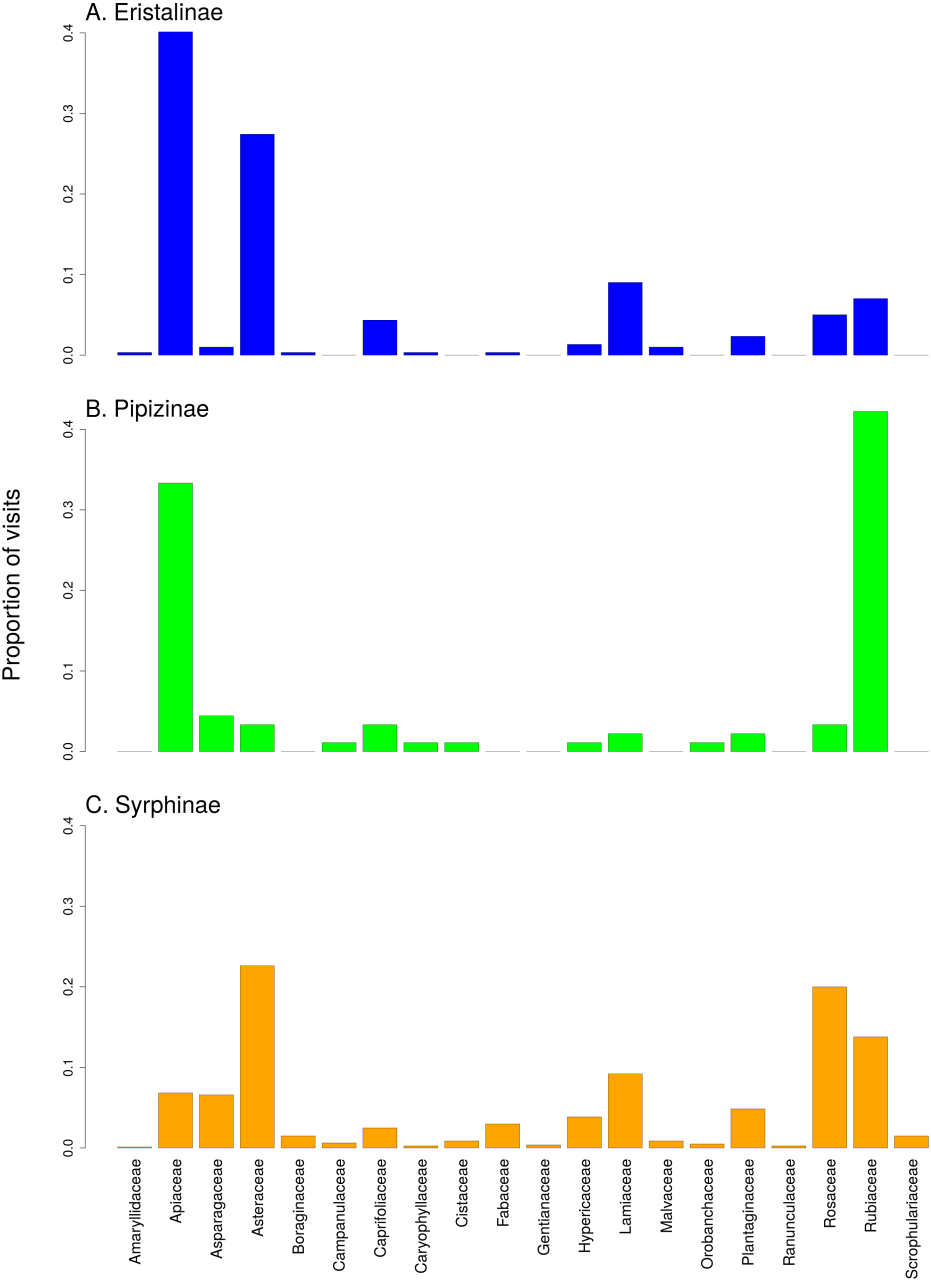
Comparison of the proportion of flower visits by the three subfamilies of Syrphidae to individual plant families. (A) Eristalinae, (B) Pipizinae, (C) Sryphinae. The bars show the proportion of observations of flower visits depending on plant family.

There was a clear differentiation between Syrphinae and Eristalinae in their flower preferences based on nonmetric multidimensional scaling (NMDS) with values of pairwise diet overlap between all pairs of species of syrhids (Fig. 4). Values of Pianka’s overlap index of individual syrphid species pairs ranged from 0 to 0.988 (mean = 0.338, Table S3), i.e., from completely different to almost identical pattern of visitation of flowers of individual plant species. We included only syrphid species with at least five observations in this analysis. The only two species of Pipizinae included in the analysis did not cluster together, unlike the other two subfamilies (Fig. 4). Additional insight into differences between the three subfamilies can be gained from a comparison of visitation frequency on plants from different families shown in Fig. 5.

There were only minor differences in preferences for flowers of different plant species between males and females. Comparison of the frequency of flower visits of males and females on individual plant species revealed a significant difference only in *Eristalis nemorum* (*χ*^2^ test for contingency tables; *χ*^2^ = 21.76, *P* = 0.0048; *P* estimated by 10,000 Monte Carlo simulations). Males of *Eristalis nemorum* visited mostly *Daucus carota*, while females visited mostly *Centaurea scabiosa* and *Achillea millefolium* (File S1). Other abundant species showed only minor differences in flower visitation, but the number of observations was low in many cases (File S1); species with <10 observations per sex were not included in these analyses.

**Figure 6:**
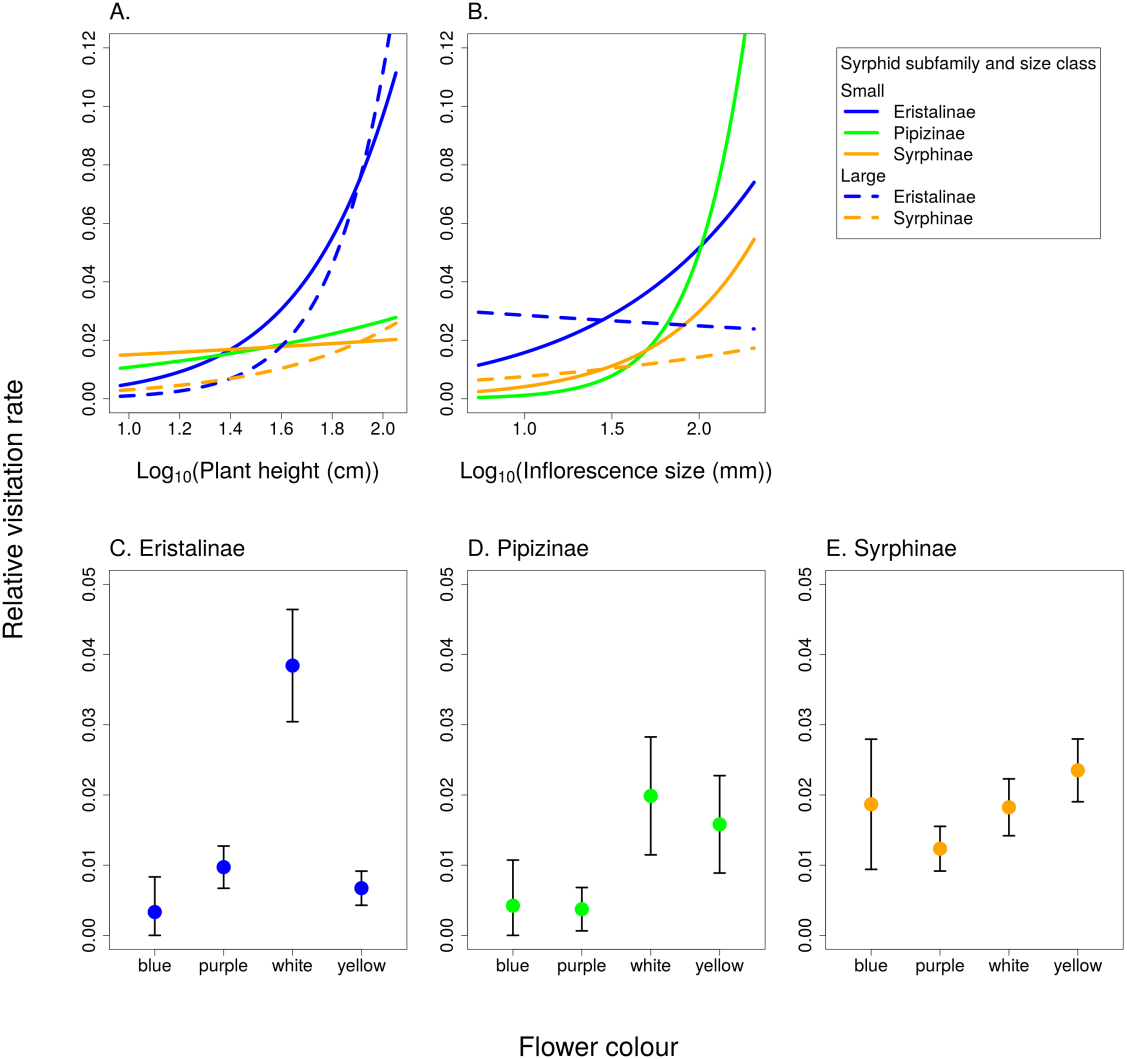
The effect of species traits on flower visitation by Syrphidae. (A) Taller plants were visited more frequently by syrphids with a slope dependent on their body size and subfamily. (B) Small syrphids visited more often plants with large inflorescences, while visitation by large syrphids was not affected by inflorescence size. (C–E) Flower colour affected visitation by the three subfamilies of syrphids differently.

Relative visitation rate of plants by individual syrphid species significantly increased with plant height, but with a different slope in syrphids from different subfamilies and different size classes (Fig. 6A, Table 3). The effect of inflorescence size also differed between small and large syrphids and between species from different subfamilies (Fig. 6B, Table 3). Specifically, relative visitation increased with inflorescence size in small syrphids from all three subfamilies, but was independent of inflorescence size in large syrphids (Fig. 6B). Flower colour also had a significant effect on visitation by syrphids, but this effect varied between the three syrphid subfamilies (Fig. 6C–6E, Table 3). Eristalinae visited mostly white flowers (Fig. 6C), Pipizinae had a similar visitation rate to yellow and white flowers (Fig. 6D), while Syrphinae showed only minor differences in visitation of flowers of different colours (Fig. 6E). When we look at the syrphid community as a whole, plants with yellow and white flowers were overall most frequently visited, with 37.7% of visits to yellow flowers and 31.3% to white flowers. Purple flowers received 26.1% visits, while blue flowers received only 4.9% of visits. In contrast to subfamily, syrphid size class did not affect the dependence of visitation on flower colour (flower colour × syrphid size class interaction, *F* = 1.27, *P* = 0.2825; Table 3).

**Table 2:** Values of the specialisation index *d*′ of male and female syrphids. Larger values of the *d*′ index correspond to more specialised flower visitation. Species where one or both sexes had <5 observations were excluded from the analysis.

	Specialisation (*d*′)	
**Species**	**Females**	**Males**
*Chrysogaster solstitialis*	0.43	0.41
*Episyrphus balteatus*	0.27	0.26
*Eristalis arbustorum*	0.29	0.28
*Eristalis nemorum*	0.27	0.26
*Lapposyrphus lapponicus*	0.17	0.23
*Melanostoma mellinum*	0.25	0.17
*Pipizella viduata*	0.27	0.28
*Sericomyia silentis*	0.34	0.29
*Sphaerophoria scripta*	0.13	0.17
*Syritta pipiens*	0.23	0.27
*Syrphus ribesii*	0.17	0.23
*Syrphus torvus*	0.16	0.26
*Syrphus vitripennis*	0.12	0.17

**Figure 7:**
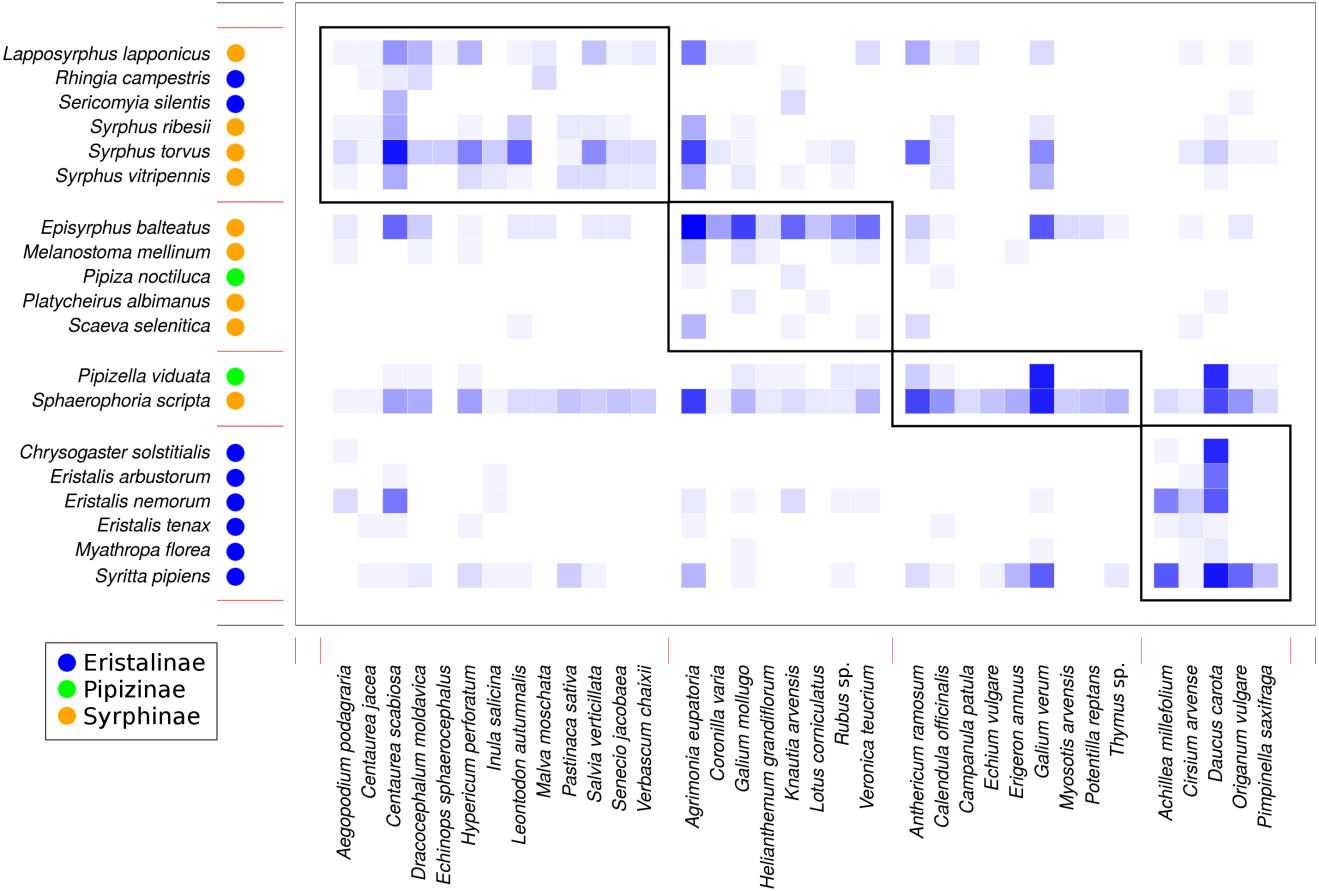
Modules detected in the plant-syrphid flower visitation network. Results of modularity analysis restricted to syrphid and plant species with at least five observations. Syrphidae are displayed in rows and plants in columns. The blue rectangles show observed interactions with more frequent interactions shown by darker colour. The three syrphid subfamilies are distinguished by coloured circles next to the species names (see legend). Modules are numbered as 1–4 from the top-left to the bottom-right.

Analysis of the structure of the plant-syrphid flower visitation network showed that the network was both modular and nested at the same time. Modularity analysis detected four modules, with most Eristalinae clustered in one module, while Syrphinae dominated two other modules, and the most generalised species, *Sphaerophoria scripta*, was grouped together with *Pipizella viduata* (Fig. 7). The association between syrphid subfamilies and the four network modules was statistically significant according to a *χ*^2^ test for contingency tables (*χ*^2^ = 16.92, *P* = 0.0069 based on 9,999 Monte Carlo simulations). Syrphid species in different modules did not differ in body length (GLM, *F* = 1.48, *P* = 0.2592) and head width (GLM, *F* = 2.66, *P* = 0.0861). In plants, the only trait significantly related to module membership was plant height (GLM, *F* = 6.57, *P* = 0.0015). Plants in modules 1 and 4 were on average taller (mean height 69 and 62 cm) than those in modules 2 and 3 (mean height 41 and 39 cm, respectively). On the other hand, plants in different modules did not differ in inflorescence size (GLM, *F* = 1.55, *P* = 0.2217) and colour (*χ*^2^ test for contingency tables, *χ*^2^ = 11.43, *P* = 0.2563 based on 9999 Monte Carlo simulations).

**Figure 8:**
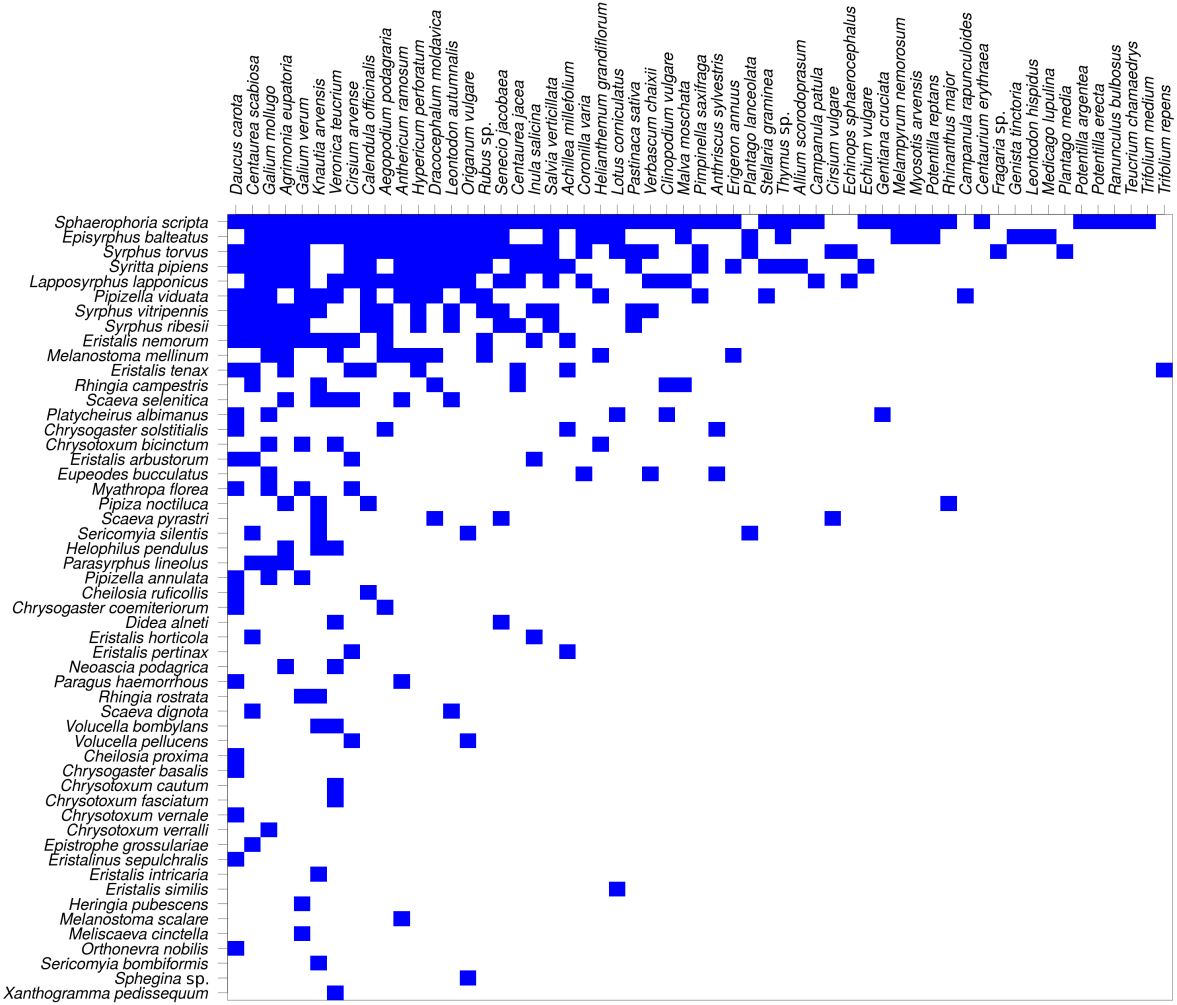
The plant-hoverfly flower visitation network was significantly nested. Syrphidae are displayed in rows and plants in columns. The blue rectangles show observed interactions.

The network was not only modular, but also nested (Fig. 8). Nestedness index was significantly different from the null model (*NODF* = 29.18, *P* < 0.007, based on 999 simulations). However, comparison of nestedness calculated for syrphids and plants separately showed that only the syrphids had significantly nested pattern of interactions (*NODF* = 19.04, *P* < 0.001), while the pattern for plants was not significantly different from the null model (*NODF* = 37.93, *P* = 0.329).

## Discussion

Flower visitation by Syrphidae was characterised by a variable degree of specialisation at the species level. Syrphids have been traditionally considered to be generalised flower visitors (Branquart & Hemptinne, 2000; Lucas et al., 2018a). We showed that not only different species fell in different positions along a gradient from more specialised to truly generalised flower visitors, but that there were also significant differences in average specialisation between the three syrphid subfamilies. The pattern of higher specialisation of Eristalinae and Pipizinae compared to the more generalised Syrphinae was clear, although our observations included only four species of Pipizinae. The values of the specialization index *d*′ ranged from 0.12 to 0.43, which is similar to previous studies based on flower visitation of hoveflies (Weiner et al., 2011; Weiner et al., 2014), but higher than reported in a recent study by Lucas et al. (2018a), who reported lower values of *d*′ based on DNA metabarcoding of pollen in 11 species of hoverflies. Still, all species we studied are fairly generalised compared to values of *d*′ reported for various other pollinators (Weiner et al., 2011; Weiner et al., 2014; Benadi et al., 2014).

**Table 3:** The effect of species traits on flower visitation by syrphids. Results of a GLM testing the dependence of relative visitation rate on species traits. Significance of all interaction terms in the model is shown.

Model term	*df*	*F*	*P*
Log_10_(plant height) × syrphid subfamily	2	4.32	0.0136
Log_10_(plant height) × syrphid size class	1	4.58	0.0325
Log_10_(inflorescence size) × syrphid subfamily	2	3.12	0.0444
Log_10_(inflorescence size) × syrphid size class	1	7.30	0.0070
Flower colour × syrphid subfamily	6	4.12	0.0004
Flower colour × syrphid size class	3	1.27	0.2825

We also found pronounced differences in relative flower preferences both at a coarse level between subfamilies, and at a finer level between species. Results of the NMDS showed that Eristalinae and Syrphinae were separated in the diagram, but also that species from the same genus clustered together, e.g., the three species of each of the genera *Eristalis* and *Syrphus* (Fig. 4). Values of pairwise overlap in flower visitation were as high as 0.98 in *Eristalis arbustorum* and *Chrysogaster solstitialis* (the maximum possible value is 1.0), and the three species of the genus *Syrphus* had overlap values between 0.83 and 0.88, which suggests that they had almost identical pattern of flower visitation. On the other hand, many species had very small values of overlap (Table S3); i.e., they visited a distinct set of plants. This is noteworthy in relation to ongoing debates about mechanisms of species coexistence. Conflicting theoretical explanations of species coexistence showed that species can coexist only if they are sufficiently different according to classic theory of limiting similarity (Hardin, 1960; MacArthur & Levins, 1967), or alternatively if they are sufficiently similar as proposed by Hubbell’s neutral theory (Hubbell, 2001). An emerging consensus is that both explanations are correct, i.e., that species can coexist if they are either sufficiently different or sufficiently similar (Scheffer & Van Nes, 2006; Sakavara et al., 2018; Scheffer, Van Nes & Vergnon, 2018). Indeed, there are examples of closely related coexisting species with different trophic niches, as well as examples where they have a very similar niche (Goulson, Lye & Darvill, 2008). In our case, we observed local coexistence of some closely related species with very high diet overlaps, which is consistent with the argument that similar species can coexist. However, caution is needed because our observations are integrated over an area of ca 2 km^2^ and a time span of more than one month. Hence, diet overlap as estimated by our flower visitation data is only one of multiple factors affecting the hoverfly community. Coexistence may be also facilitated by differences in microhabitat choice leading to small-scale spatial structuring of the community (Janovský et al., 2013) or differences in timing of activity during the day (Gilbert, 1980). More data would be needed to address these issues.

Trait-based analysis of relative visitation rate of flowers by individual syrphid species showed that syrphid subfamilies differed in flower colour preferences. Eristalinae appeared to prefer white flowers, while Syrphinae were relatively indiscriminate in the colour of flowers they visited (Fig. 6). Previous studies conducted with a smaller number of species found that hoverflies visit mostly white or yellow flowers (Haslett, 1989a; Sutherland, Sullivan & Poppy, 1999), with some exceptions, such as *Rhingia campestris* with a preference for blue flowers (Haslett, 1989a). Our results demonstrating differences between subfamilies are in line with a previous observation that out of a group of six species, *Episyrphus balteatus* from the subfamily Syrphinae was the least selective species towards flower colour, while several species from the subfamily Eristalinae were more selective (Haslett, 1989a). There is not enough known about the visual system of different species, but it is likely that interspecific differences in visitation of flowers of different colours represent foraging preferences rather than differences in the visual system which seems to be quite uniform among Diptera (Lunau, 2014). The dominant flower colour represents probably a relatively long-range visual signal, while other cues may be used when the hoverfly approaches the flower (Lunau & Wacht, 1994). Further research is needed to explore the role of subtler differences in flower colour than we considered here, in particular the role of UV reflectance, which we did not measure.

Based on our results, Eristalinae and Pipizinae showed a stronger response to all plant traits, i.e., flower colour, plant height and inflorescence size, compared to Syrphinae. Taken together, these results highlight the differences in average specialisation level between the generalised Syrphinae on one side and more specialised Eristalinae and Pipizinae on the other side. Interestingly, Moquet et al. (2018) found that they could split hoverflies of Belgian heathlands according to an analysis of several life-history and ecological traits into two distinct groups roughly corresponding to the two dominant subfamilies, Eristalinae and Syrphinae. Our detailed analysis of flower visitation provides additional evidence of ecological differences between the syrphid subfamilies.

Apart from phylogenetic relatedness at the subfamily level, we found body size to be an important trait modifying the responses of syrphid relative visitation rate to selected plant traits. Flower colour was related mostly to phylogenetic relatedness, while plant height and inflorescence size were related also to syrphid body size. Small syrphids preferred large inflorescences, which may be advantageous because they contain a large amount of resources clustered in one place (Akter, Biella & Klecka, 2017). Another trait that has been evaluated previously is the relationship between corolla depth and proboscis length. Some previous studies showed a positive correlation between the average depths of flowers and proboscis length or length/width ratio in bees (Stang, Klinkhamer & Van Der Meijden, 2006; Stang et al., 2009) as well as hoverflies (Gilbert, 1981; Branquart & Hemptinne, 2000). We did not test this relationship mostly because we did not distinguish nectar and pollen feeding. Even species with a short proboscis are regularly visiting long-spurred flowers to feed on pollen and can even lick nectar at the entrance to the spur without being able to reach deep inside (Vlašánková et al., 2017). Proper analysis of a morphological fit between the flowers and flower visitors would thus require a more detailed data on mechanisms of feeding by individual species and on morphology of both the insects and the flowers.

Network modularity (Olesen et al., 2007) partly reflected these patterns, because we found that the plant-hoverfly flower visitation network could be partitioned into four modules, which were significantly different in the proportion of the three subfamilies of Syrphidae. Plants were grouped partly according to their height. The two modules containing Eristalinae had plants on average 50% taller then the other two modules, which fits well with results of our analyses of the relationship between species traits and visitation (see Fig. 6). Hence, modularity of the network was related to phylogeny (syrphids) and species traits (plants) as expected based on previous studies on plant–pollinator networks (Carstensen, Sabatino & Morellato, 2016; Dormann, Fründ & Schaefer, 2017). However, our more detailed analyses on the role of species traits for flower visitation also identified relationships which were not reflected in the modular pattern, e.g., the role of flower colour and syrphid body size. This is likely because the modularity analysis turns quantitative data on flower visitation into categorical data (membership in modules), which leads to the loss of information.

Nestedness analysis showed that the syrphid flower visitation was significantly nested, i.e., that more specialised species visited mostly flowers of plants which were a subset of those visited by more generalised species, which is a typical pattern in plant-flower visitor networks (Bascompte et al., 2003; Fortuna et al., 2010). However, nestedness of the plants did not differ from the null model, so the nestedness pattern was asymmetric. This is likely because the plants were visited by a range of other insects, not only hoverflies, so the network we analysed was incomplete from the plants’ point of view.

Despite the patterns we found at the interspecific level, we detected very little differences in flower visitation by males and females of species sufficiently abundant to allow such comparison. Both the level of specialisation and the relative visitation rates to individual plant species were very similar in males and females in most cases. Similarly, Lucas et al. (2018b) found no effect of sex on individual-level specialisation in several *Eristalis* species and Sutherland, Sullivan & Poppy (1999) found that males and females of *Episyrphus balteatus* showed very similar flower colour preferences. However, we did not distinguish nectar and pollen consumption during our observations, so we cannot rule out a possible difference between sexes in pollen vs. nectar feeding. Indeed, several previous studies reported that females of hoverflies feed on pollen more frequently than males (Gilbert, 1981; Haslett, 1989b; Hickman, Lövei & Wratten, 1995), probably because proteins from pollen are necessary for egg development. Males thus often feed less on pollen and more on nectar which serves mostly as a source of energy for their active lifestyle, because they are usually more active than females and spend a large amount of time by hovering (Haslett, 1989b). However, no significant difference in pollen consumption between males and females was found in a few other species, so the generality of this patterns is unclear (Irvin et al., 1999).

We should point out that our data have some limitations. First, we observed flower visitation by syrphids over an area of ca 2 km^2^ over a period of over 5 weeks. Species interactions can vary both in time and space depending on phenology and small-scale spatial distribution of both plants and flower visitors. We do not have enough data to explore such variation here, but it is an important avenue for future research (Carstensen, Sabatino & Morellato, 2016; Spiesman & Gratton, 2016; Tylianakis & Morris, 2017). Second, the spatial and temporal scale of observations prevented us from collecting detailed information about the abundance of individual plants and their value as sources of nectar and pollen. Including such information would be necessary for a more mechanistic analysis of resource use by flower visitors (Hicks et al., 2016). Third, we restricted our attention to insect-pollinated plants. However, some syrphids can feed also on pollen of wind-pollinated plants either by visiting flowers of grasses, sedges, trees, etc., or by eating pollen accumulated on the surface of leaves (Ssymank & Gilbert, 1993; Saunders, 2017). A better understanding of this phenomenon, as well as possible use of other food sources, will be important to achieve better understanding of foraging biology of hoverflies.

In conclusion, we found that hoverflies in a typical central European grassland varied in their use of floral resources depending on their relatedness (differences in flower visitation between subfamilies) and body size. We identified three plant traits which could partly explain differences in visitation by different hoverfly species: plant height, inflorescence size, and flower colour. However, there is still a range of questions to be addressed by future studies, such as whether differences in flower visitation stem from active choice by the foraging hoverflies and whether they could be explained mechanistically, e.g., using optimal foraging theory.

##  Supplemental Information

10.7717/peerj.6025/supp-1 File S1 Visitation of flowers of individual plant species by selected species of hoverflies, separately by males and females

10.7717/peerj.6025/supp-2 Table S1 Raw data on flower visitation by hoverflies The dataset contains all observations of flower visits. Each hoverfly individual is recorded in a separate row and information about its identity, sex, sampling location, and plant species on whose flower it was collected is provided.

10.7717/peerj.6025/supp-3 Table S2 The complete plant-hoverfly flower visitation network The network is shown as a matrix with hoverflies in rows and plants in columns. Number of flower visits recorded is provided for each plant-hoverfly species combination. The first column provides hoverfly species ID number and the first raw provides plant species ID number used in Fig. 1 in the main text.

10.7717/peerj.6025/supp-4 Table S3 Values of diet overlap for all pairs of hoverfly species We show values of the Pianka’s niche overlap index calculated from flower visitation data. Species of hoverflies with <5 records were excluded from the calculations.

10.7717/peerj.6025/supp-5 Table S4 A summary of plant species trait measurements Mean value of plant height and inflorescence size, and flower colour is provided for all plant species visited by hoverflies during our study.

## Additional Information and Declarations

### Data Availability

The following information was supplied regarding data availability: The raw data are included as Supplemental Files.
