# Supplementary material for: Flower visitation by hoverflies (Diptera: Syrphidae) in a temperate plant-pollinator network
Source: PeerJ. 2018 Dec 3;6:e6025. doi: 10.7717/peerj.6025 (PMC6282941; doi:10.7717/peerj.6025)
Supplement: File S1 [file peerj-06-6025-s001.pdf]

Flower visitation by hoverflies (Diptera: Syrphidae) in a temperate plant-pollinator network
Supplementary File 1

Jan Klecka[1], Jiří Hadrava[1,2], Paolo Biella[1,3], and Asma Akter[1,3]

[1]**Czech Academy of Sciences, Biology Centre, Institute of Entomology, České Budějovice, Czech Republic**
[2]**Department of Zoology, Faculty of Science, Charles University, Prague, Czech Republic**
[3]**Department of Zoology, Faculty of Science, University of South Bohemia, České Budějovice, Czech Republic**

Corresponding author:
Jan Klecka

Email address: jan.klecka@entu.cas.cz

VISITATION DATA FOR SELECTED SPECIES

We show the number of observations for females and males of the ten most abundant species collected on flowers of individual plant species. We tested the difference in the proportion of visits to different plant species between males and females using a χ^2 test for contingency tables. The values of P were estimated by 10000 Monte Carlo simulations in all cases. Complete visitation data for all species are available in Supplementary Table 1.

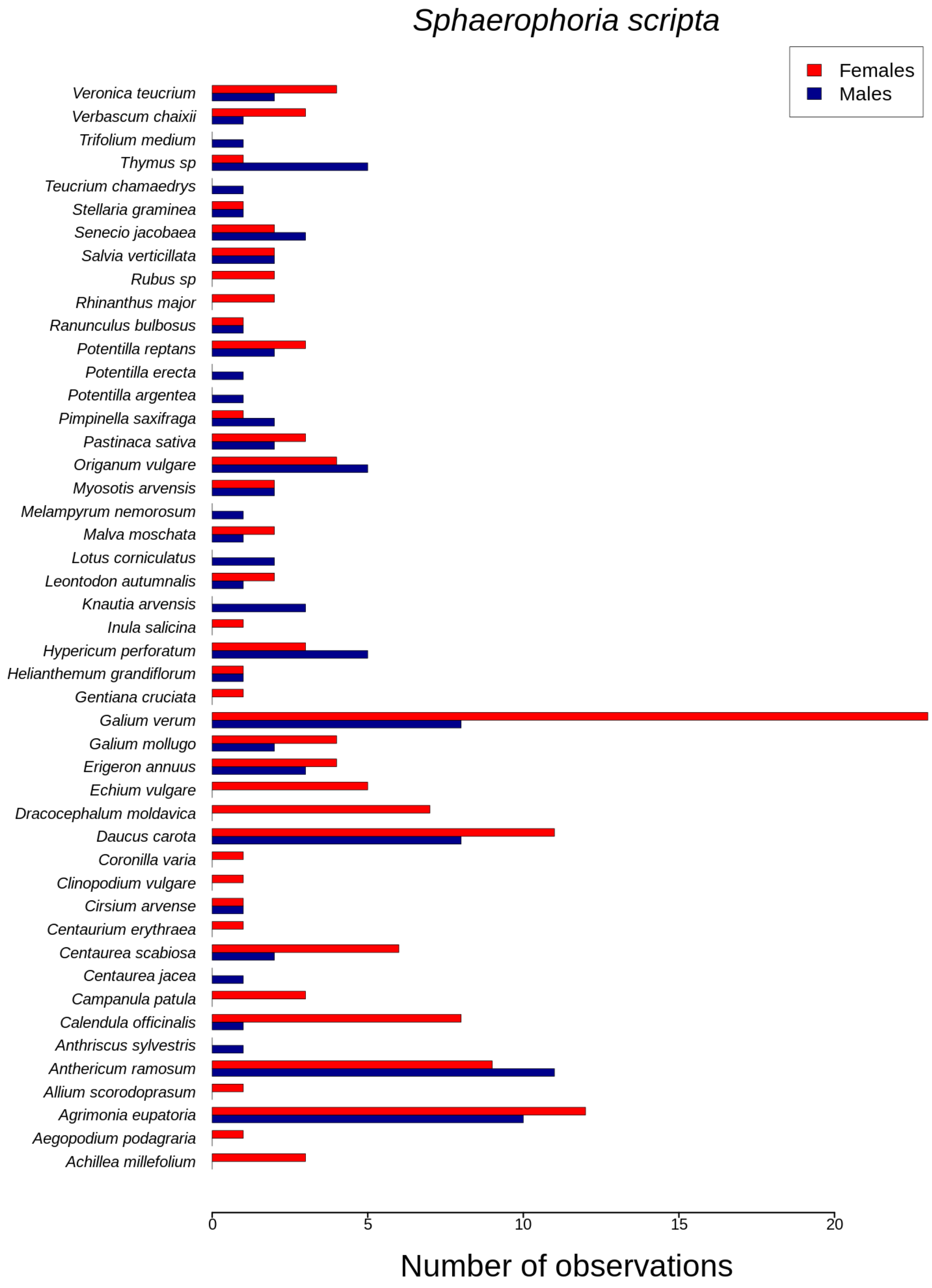

Figure 1. Flower visitation by *Sphaerophoria scripta*. Total number of observations was 236, 142 for females and 94 for males. The difference in the proportion of visits to different plant species between males and females was not significant ($\chi^2 = 56.83$, $P = 0.0720$).

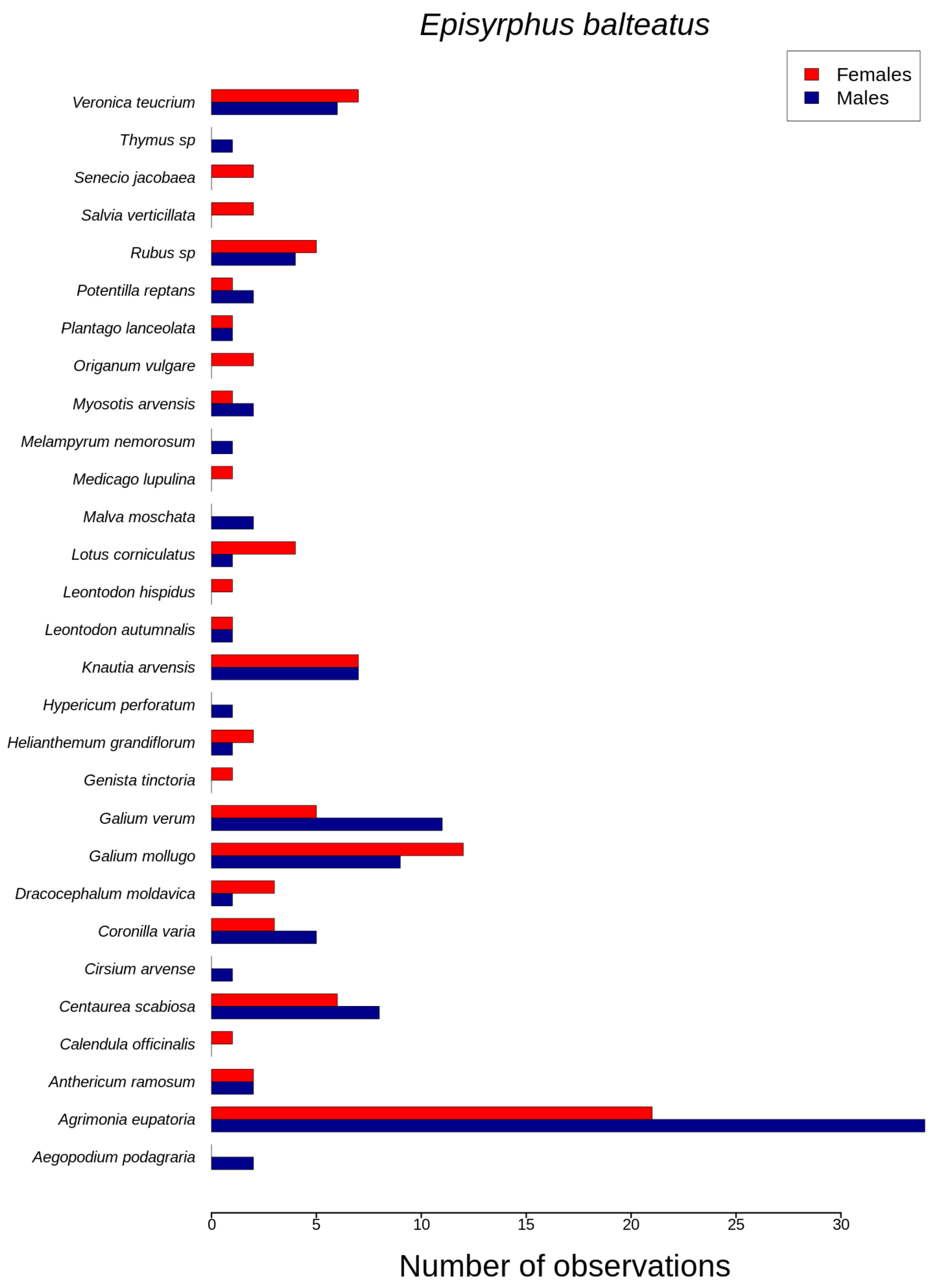

Figure 2. Flower visitation by *Episyrphus balteatus*. Total number of observations was 194, 91 for females and 103 for males. The difference in the proportion of visits to different plant species between males and females was not significant ($\chi^2 = 27.89$, $P = 0.4931$).

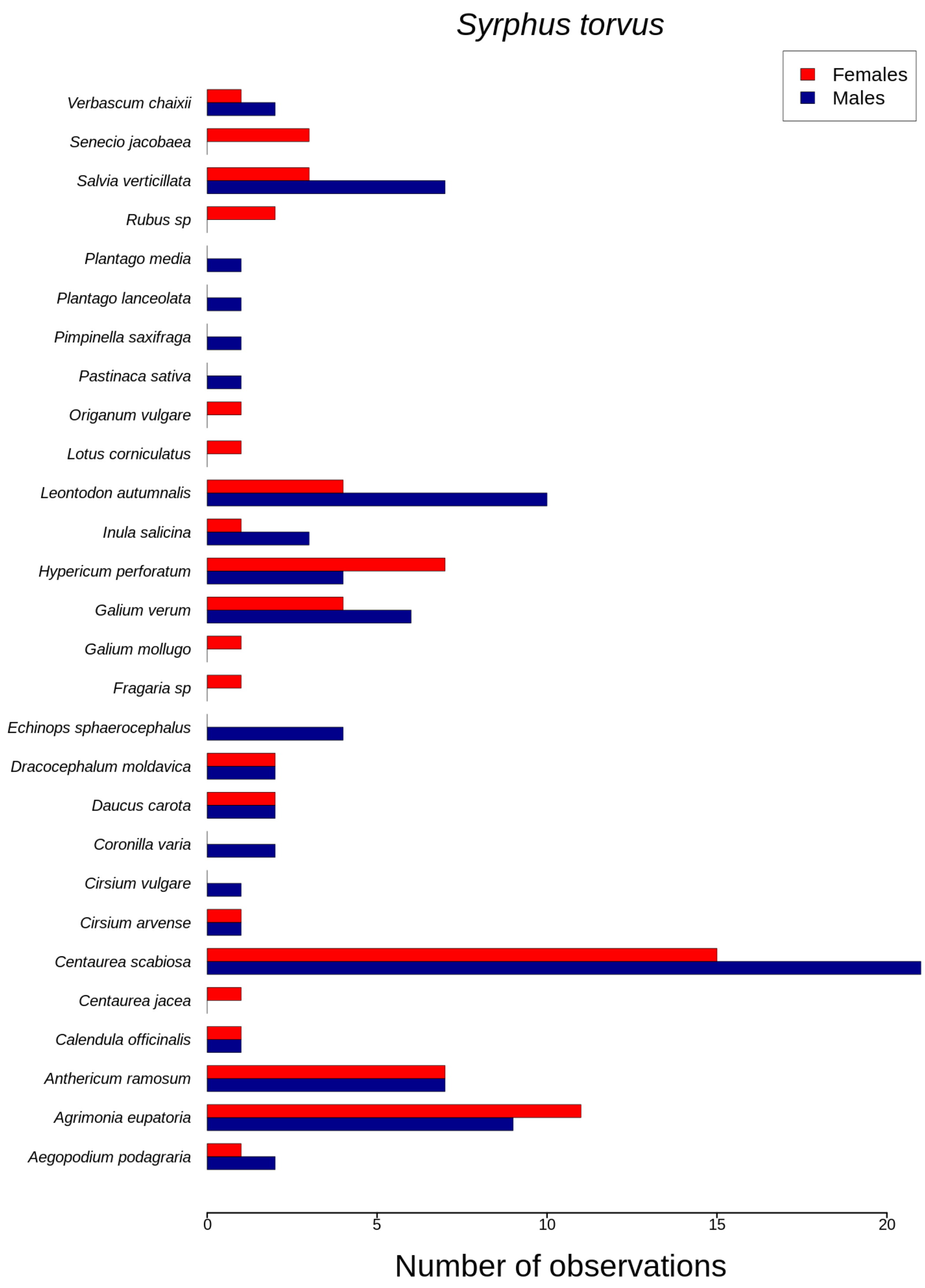

Figure 3. Flower visitation by *Syrphus torvus*. Total number of observations was 158, 70 for females and 88 for males. The difference in the proportion of visits to different plant species between males and females was not significant ($\chi^2 = 27.56$, $P = 0.4372$).

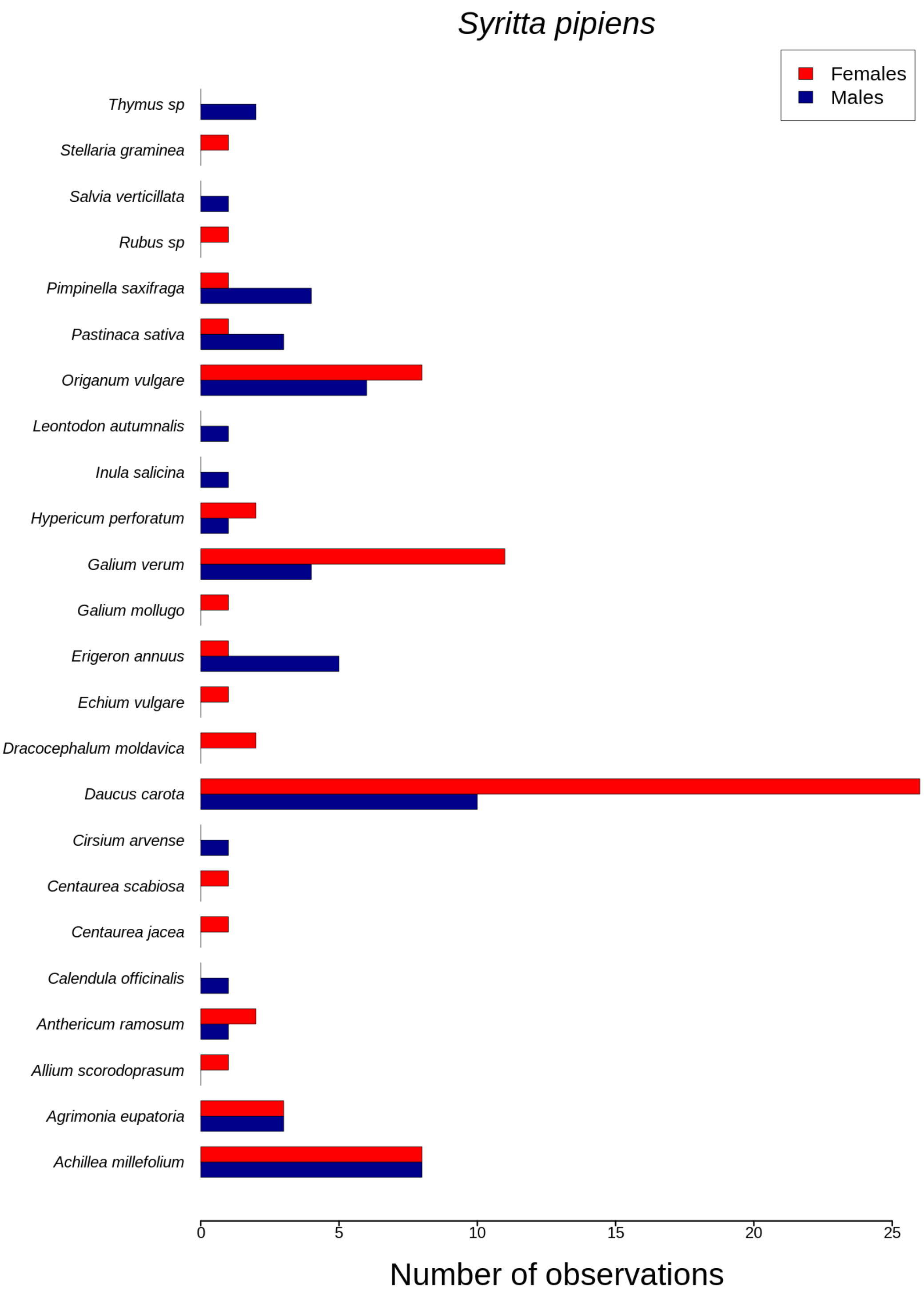

Figure 4. Flower visitation by *Syritta pipiens*. Total number of observations was 124, 72 for females and 52 for males. The difference in the proportion of visits to different plant species between males and females was not significant according to a χ^2 test for contingency tables ($\chi^2 = 30.36, P = 0.0586$), but females visited *Daucus carota* and *Galium verum* with much higher frequency than males.

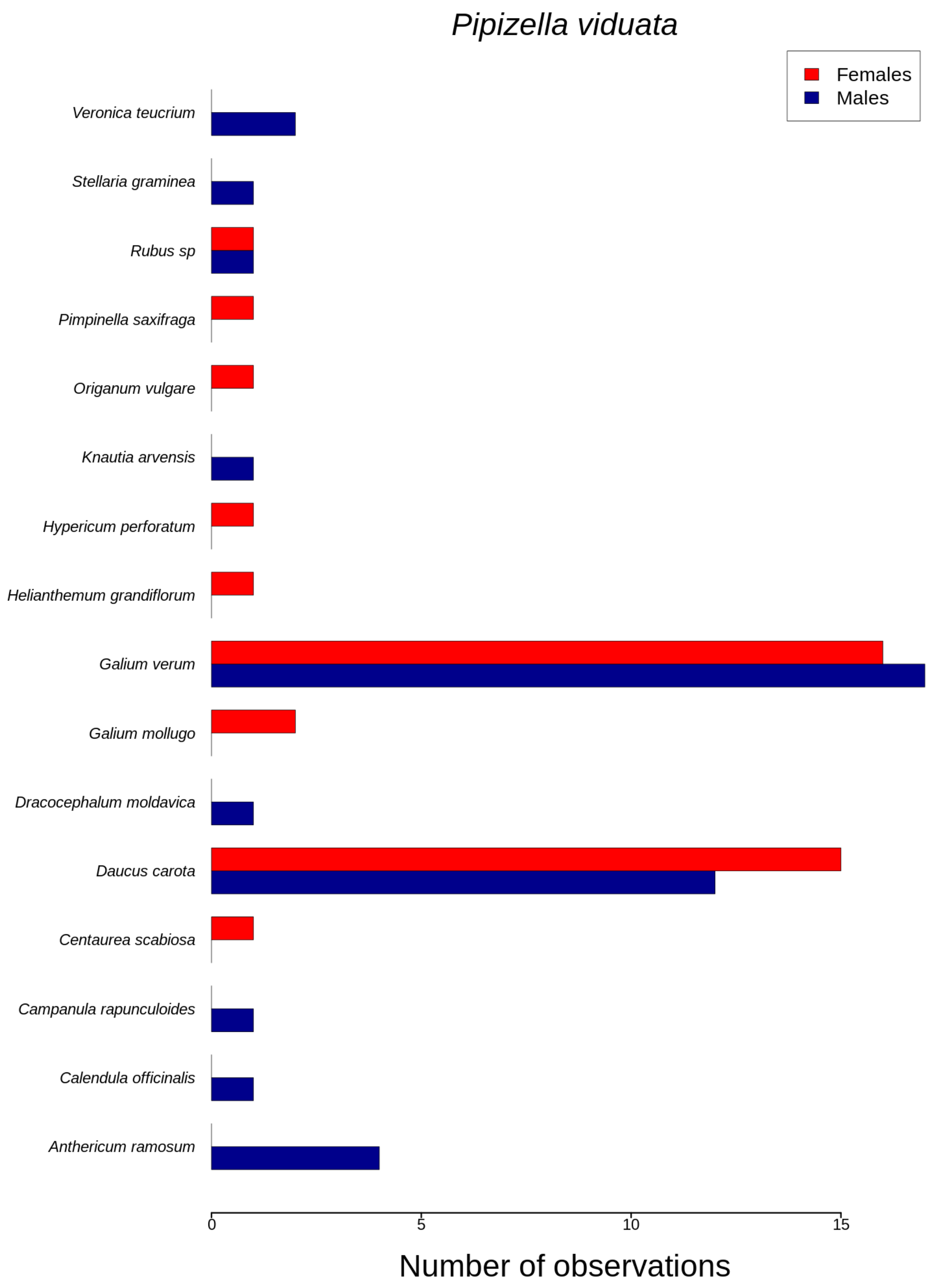

Figure 5. Flower visitation by *Pipizella viduata*. Total number of observations was 80, 39 for females and 41 for males. The difference in the proportion of visits to different plant species between males and females was not significant ($\chi^2 = 18.33$, $P = 0.1188$).

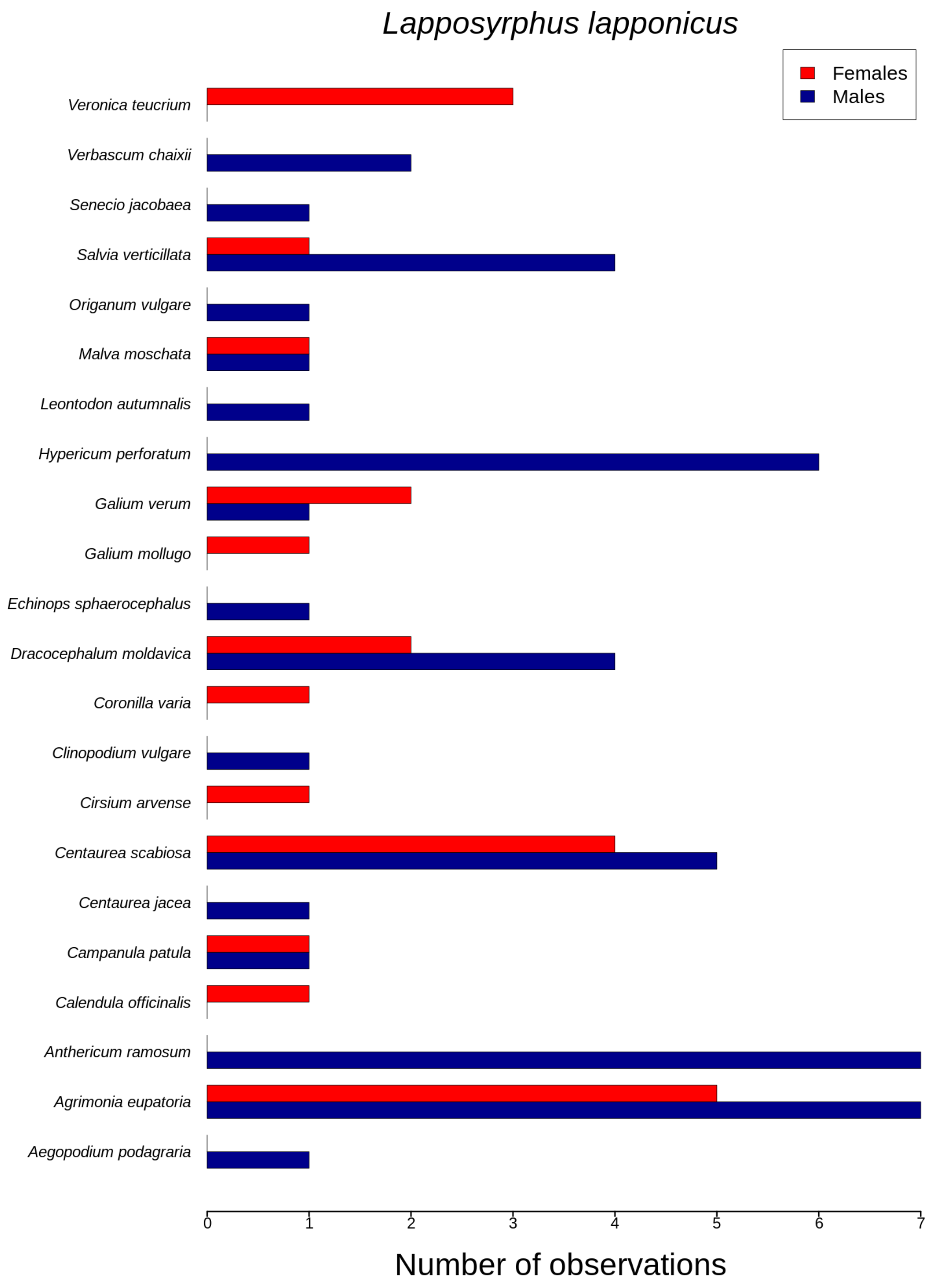

Figure 6. **Flower visitation by *Lapposyrphus lapponicus*.** Total number of observations was 68, 23 for females and 45 for males. The difference in the proportion of visits to different plant species between males and females was not significant ($\chi^2 = 28.05$, $P = 0.0703$), although it is notable that only males were collected on *Anthericum ramosum* and *Hypericum perforatum* (7 and 6 individuals, respectively).

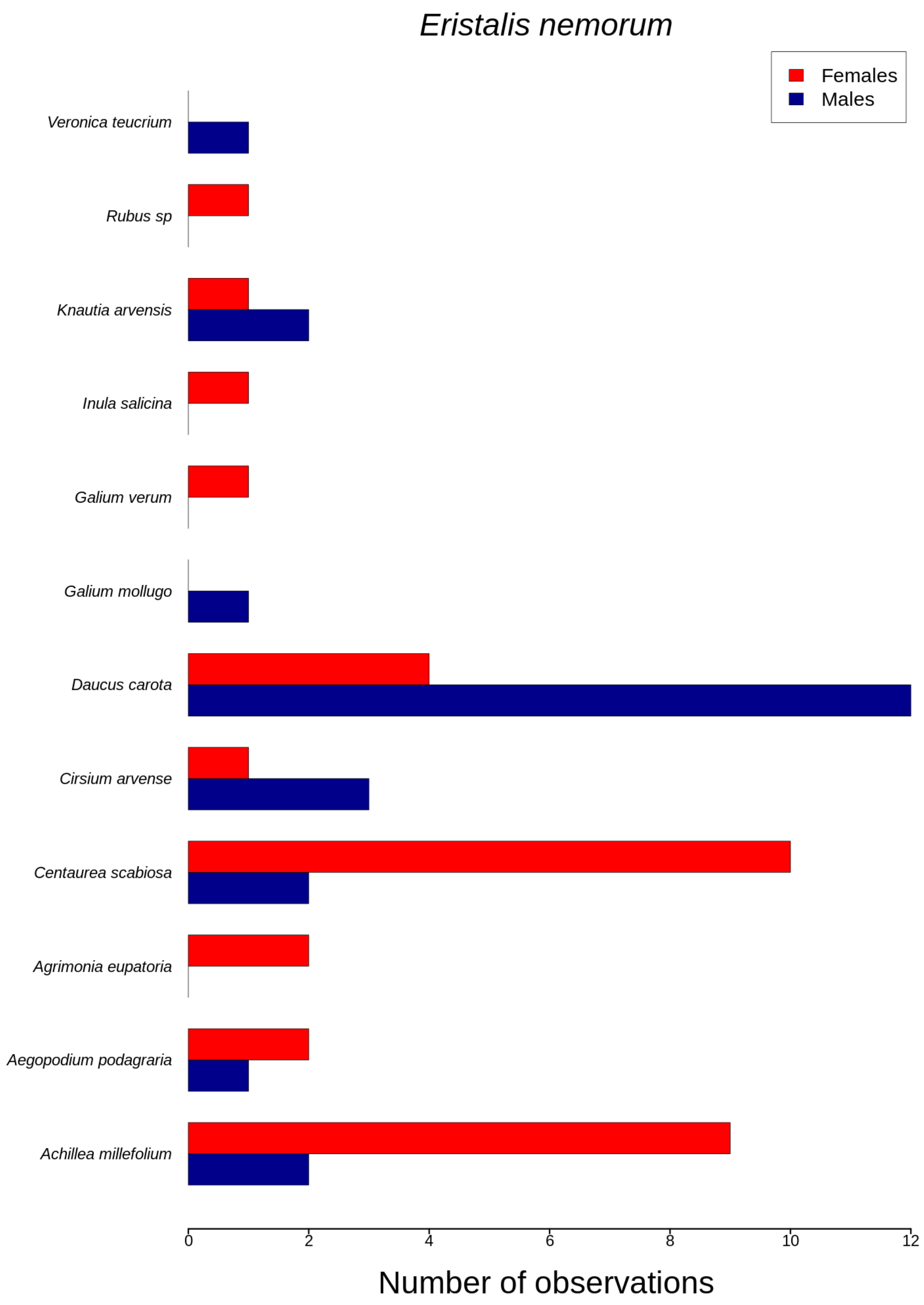

Figure 7. Flower visitation by *Eristalis nemorum*. Total number of observations was 56, 32 for females and 24 for males. The proportion of visits to different plant species was significantly different between males and females ($\chi^2 = 21.756$, $P = 0.0043$).

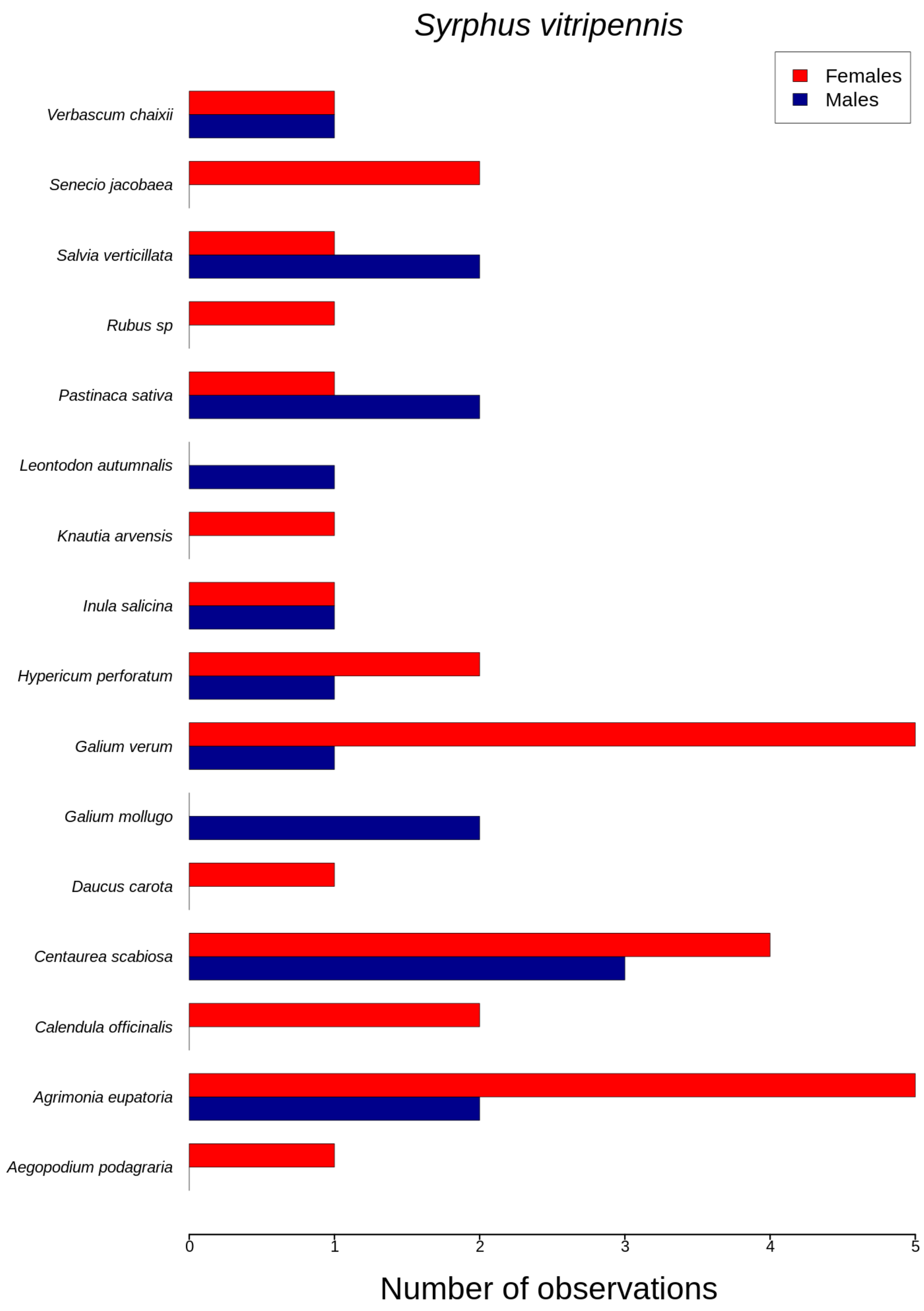

Figure 8. **Flower visitation by *Syrphus vitripennis*.** Total number of observations was 44, 28 for females and 16 for males. The difference in the proportion of visits to different plant species between males and females was not significant ($\chi^2 = 13.85$, $P = 0.6416$).

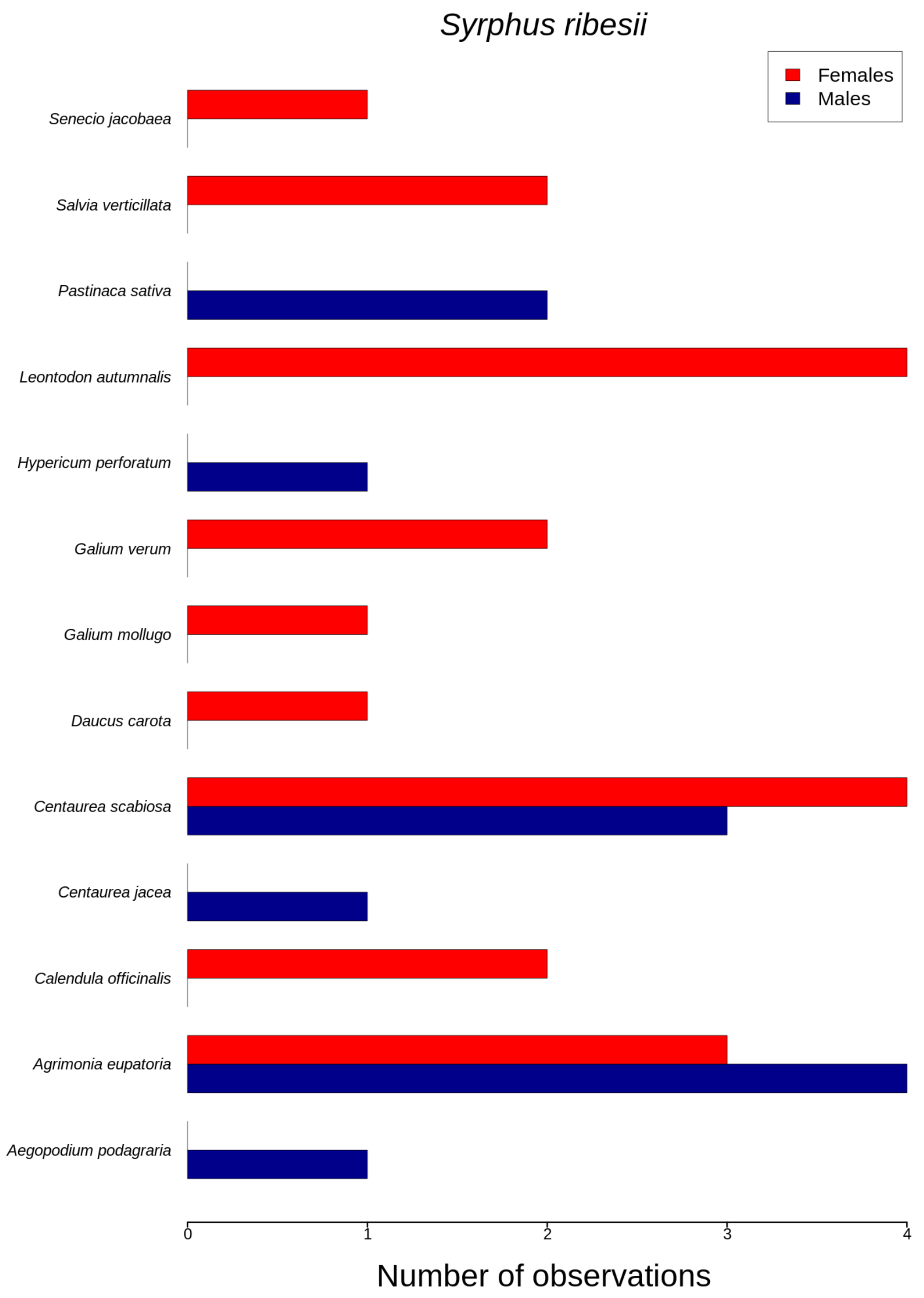

Figure 9. Flower visitation by *Syrphus ribesii*. Total number of observations was 32, 20 for females and 12 for males. The difference in the proportion of visits to different plant species between males and females was not significant ($\chi^2 = 17.37$, $P = 0.0631$).

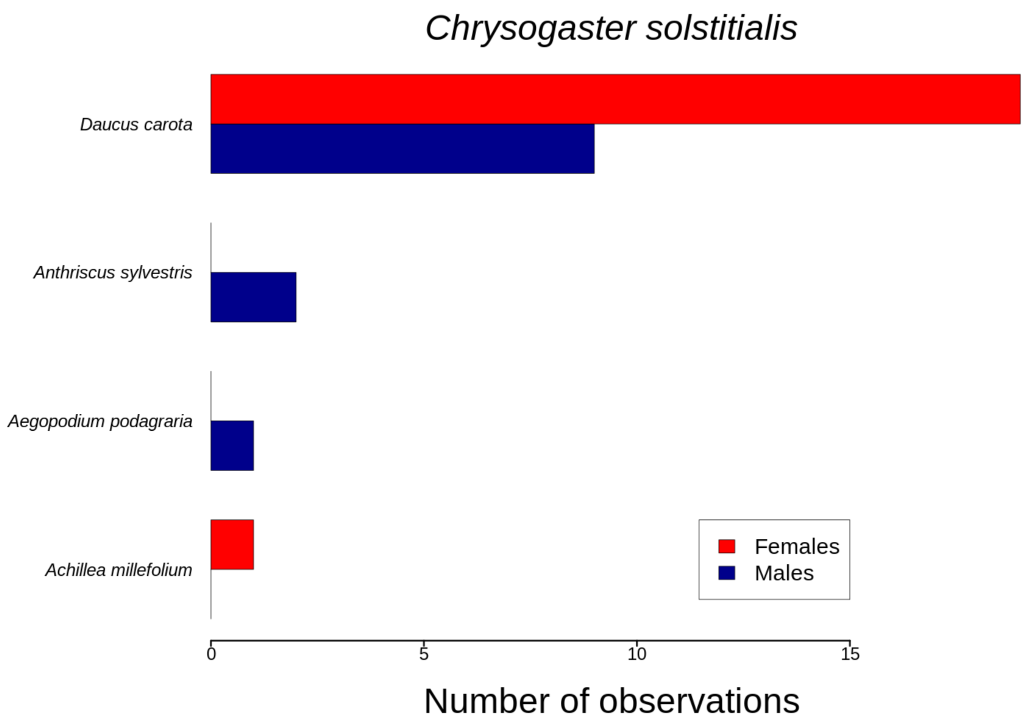

Figure 10. Flower visitation by *Chrysogaster solstitialis*. Total number of observations was 32, 20 for females and 12 for males. The difference in the proportion of visits to different plant species between males and females was not significant ($\chi^2 = 5.94$, $P = 0.0764$).
